# Preoperative exercise induces endothelial progenitor cell mobilisation in patients undergoing major surgery – A prospective randomised controlled clinical proof-of-concept trial

**DOI:** 10.1016/j.heliyon.2022.e10705

**Published:** 2022-09-23

**Authors:** Claus Juergen Bauer, Michael Findlay, Christina Koliamitra, Philipp Zimmer, Volker Schick, Sebastian Ludwig, Geoffrey C. Gurtner, Bernhard Riedel, Robert Schier

**Affiliations:** a Department of Internal Medicine—Oncology, Hematology and Rheumatology, University Hospital Bonn, Bonn, Germany; b Department of Surgery, Division of Cancer Surgery, Peter MacCallum Cancer Centre, Melbourne, Australia; c Institute for Cardiovascular Research and Sports Medicine, German Sports University Cologne, Cologne, Germany; d Institute of Sports and Sports Medicine, TU Dortmund University, Dortmund, Germany; e Department of Anaesthesiology and Intensive Care Medicine, Faculty of Medicine and University Hospital Cologne, University of Cologne, Cologne, Germany; f Department of Obstetrics and Gynaecology, Faculty of Medicine and University Hospital Cologne, University of Cologne, Cologne, Germany; g Department of Surgery, Division of Plastic and Reconstructive Surgery, Stanford University School of Medicine, Stanford, USA; h Department of Anaesthetics, Perioperative Medicine and Pain Medicine, Peter MacCallum Cancer Centre, Melbourne, Australia

**Keywords:** Endothelial progenitor cells, Preoperative exercise, Prehabilitation, Cluster-based analysis, Cell mobilisation, Postoperative complications

## Abstract

**Introduction:**

Prehabilitation is increasingly recognised as a therapeutic option to reduce postoperative complications. Investigating the beneficial effects of exercise on cellular mechanisms, we have previously shown that a single episode of exhaustive exercise effectively stimulates endothelial progenitor cells (a cell population associated with vascular maintenance, repair, angiogenesis, and neovascularization) in correlation with fewer postoperative complications, despite the ongoing debate about the appropriate cell surface marker profiles of these cells (common phenotypical definitions include CD45dim, CD133+, CD34+ and/or CD31+). In order to translate these findings into clinical application, a feasible prehabilitation programme achieving both functional and cellular benefits in a suitable timeframe to expedite surgery is necessary.

**Objective:**

The objective of this study was to test the hypothesis that a four-week prehabilitation programme of vigorous-intensity interval exercise training is feasible, increases physical capacity (primary outcome) and the circulatory number of endothelial progenitor cells within peripheral blood.

**Methods:**

In this unblinded, parallel-group, randomised controlled proof-of-concept clinical trial (German Clinical Trial Register number: DRKS00000527) conducted between 01^st^ December 2014 and 30^th^ November 2016, fifteen female adult patients scheduled for incontinence surgery with abdominal laparotomy at the University Hospital Cologne were allocated to either an exercise (n = 8, exclusion of 1 patient, analysed n = 7) or non-exercise group (n = 7, exclusion of 1 patient, analysed n = 6). The exercise group's intervention consisted of a vigorous-intensity interval training for four weeks preoperatively. Cardiopulmonary Exercise Testing accompanied by peripheral blood collection was performed before and after the (non-)training phase. Cellular investigations were conducted by flow cytometry and cluster-based analyses.

**Results:**

Vigorous-intensity interval training over four weeks was feasible in the exercise group (successful completion by 8 out of 8 patients without any harms), with significant improvements in patients' functional capacity (increased oxygen uptake at anaerobic threshold [intervention group mean + 1.71 ± 3.20 mL/min/kg vs. control group mean −1.83 ± 2.14 mL/min/kg; p = 0.042] and peak exercise [intervention group mean + 1.71 ± 1.60 mL/min/kg vs. control group mean −1.67 ± 1.37 mL/min/kg; p = 0.002]) and a significant increase in the circulatory number of endothelial progenitor cells (proportionate CD45dim/CD14dim/CD133+/CD309+/CD34+/CD31 + subpopulation within the circulating CD45-pool [p = 0.016]).

**Conclusions:**

We introduce a novel prehabilitation concept that shows effective stimulation of an endothelial progenitor cell subpopulation within four weeks of preoperative exercise, serving as a clinical cell-mediated intervention with the aim to reduce surgical complications.

**Funding:**

Institutional funding. DFG (German Research Foundation, 491454339) support for the Article Processing Charge.

## 1 Introduction

Surgery is required for more than one-third of the worldwide burden of disease [1], with millions of surgical procedures performed each year globally [2, 3]. Unfortunately, as many as half of the patients having major surgery suffer postoperative complications, and death within 30 days after surgery is the third leading cause of death globally, after death from cardiovascular disease and stroke [4]. Postoperative complications nearly double the cost of providing surgical care [5]. Therefore, the preoperative period is an ideal window of opportunity for therapeutic interventions to positively influence patients’ modifiable risk factors, including poor functional capacity, anaemia, malnutrition, and smoking.

Impaired circulatory and hematopoietic function is closely associated with poor postoperative outcomes. Preoperative anemia is associated with increased morbidity and mortality in patients undergoing major surgery [6]. Newer research has also brought the attention to the association of bone marrow-derived lineages other than erythrocytes with postoperative complication rates. A particular focus has been placed on endothelial progenitor cells (EPC), which have been repeatedly linked to vascular maintenance, repair, angiogenesis, and neovascularization by various publications [7, 8, 9, 10]. While measuring the EPC recruitment, in response to single-episode exhaustive exercise, may help risk-stratify patients before surgery [11], we need to investigate further the direct therapeutic effects of this regenerative cellular response on reducing postoperative complications. This will then lead to strategies to optimise progenitor cell recruitment in surgical patients to reduce the risk of postoperative complications. Preoperative exercise is one such strategy. Exercise can play an important role, as we observed that single-episode exhaustive exercise has a measurable effect on endothelial progenitor cell recruitment into the peripheral circulation [11]. The degree to which patients mobilised EPCs with such a stimulus was predictive of their postoperative complication rates (better recruitment was associated with fewer complications) [11].

In parts also referring to EPCs, a number of studies have shown the importance of exercise for microvascular health [12, 13, 14, 15, 16, 17]. However, the mechanism of endothelial repair through EPCs and its role for microvascular health is still not fully understood. Clearly evident is, that in septic patients, lack of EPC mobilisation is associated with poor microvascular function and higher mortality [18, 19]. A hypothesis that is discussed in the literature is that EPCs migrate from the bone marrow to the endothelium and settle there as part of a "homing" process. Whether these cells are capable of fully restoring endothelial function remains unclear [20].

The optimal prehabilitation regimen in order to access the beneficial potential of EPC recruitment within a suitable preoperative timeframe has not yet been defined.

In this proof-of-concept pilot study, we tested the hypothesis that a four-week prehabilitation programme of vigorous intensity interval exercise training is feasible, increases physical capacity and the circulatory number of endothelial progenitor cells within peripheral blood.

## 2 Materials and methods

### 2.1 Study design and population

Fifteen adult female patients, scheduled for gynaecological incontinence surgery with abdominal laparotomy at the University Hospital Cologne, were enrolled prospectively in this proof-of-concept clinical trial after giving written informed consent. The study was registered in the German Clinical Trial Register (DRKS00000527) in accordance with the declaration of Helsinki and received ethical approval under Institutional Review Board #13–274. Initially, this proof-of-concept trial aimed to include 20 patients (the deviation from the 100 patients target at initial trial registration is explained by the rationale to conduct a proof-of-concept study first when transitioning from a single episode of exhaustive exercise, which was the focus of investigation in our previous work, to a four-week vigorous-intensity training schedule preoperatively. In consequence, the primary endpoint shifted to the effect of the applied four-week preoperative vigorous intensity interval training programme on physical capacity in the sense of proving feasibility of the suggested prehabilitation concept and evaluating its efficacy potential).

Trial participation eligibility required a scheduled surgery date for the above-mentioned intervention, female sex, an age over 18 years and written informed consent (inclusion criteria). Contrary to initial trial registration, the inclusion criteria was narrowed to gynaecological patients only because after the trial registration we realised that orthopaedic patients have large difficulties to perform exercise preoperatively with lots of their pathologies preventing them from exercising to exertion. This negative effect had been underestimated at the time of the trial registration. In addition, the surgery selection for this study needed to be made in consideration of the usual preoperative lead time to enable the implementation of a four-week preoperative training in the first place. With the intention to ensure homogeneity of the limited-size patient population, ultimately only a single, specific surgical technique for incontinence surgery was selected. With these decisions in mind and in order not to narrow the recruitable population even further, the exclusive focus on the subpopulation of patients with metabolic syndrome (according to International Diabetes Federation Criteria)—even if more promising with regards to potential results of this study—was therefore abandoned.

Exclusion criteria included a recent history (< three months prior to screening) of myocardial infarction, new or unstable angina, venous thromboembolism or chronic deep vein thrombosis, cerebrovascular accident or transit ischaemic attacks, ongoing pregnancy, scheduled surgery too early to enable preoperative trial participation, and the inability to exercise above anaerobic threshold (self-reported, evident from patient history, or detected by first Cardiopulmonary Exercise Testing after initial trial participation).

**Figure 1:**
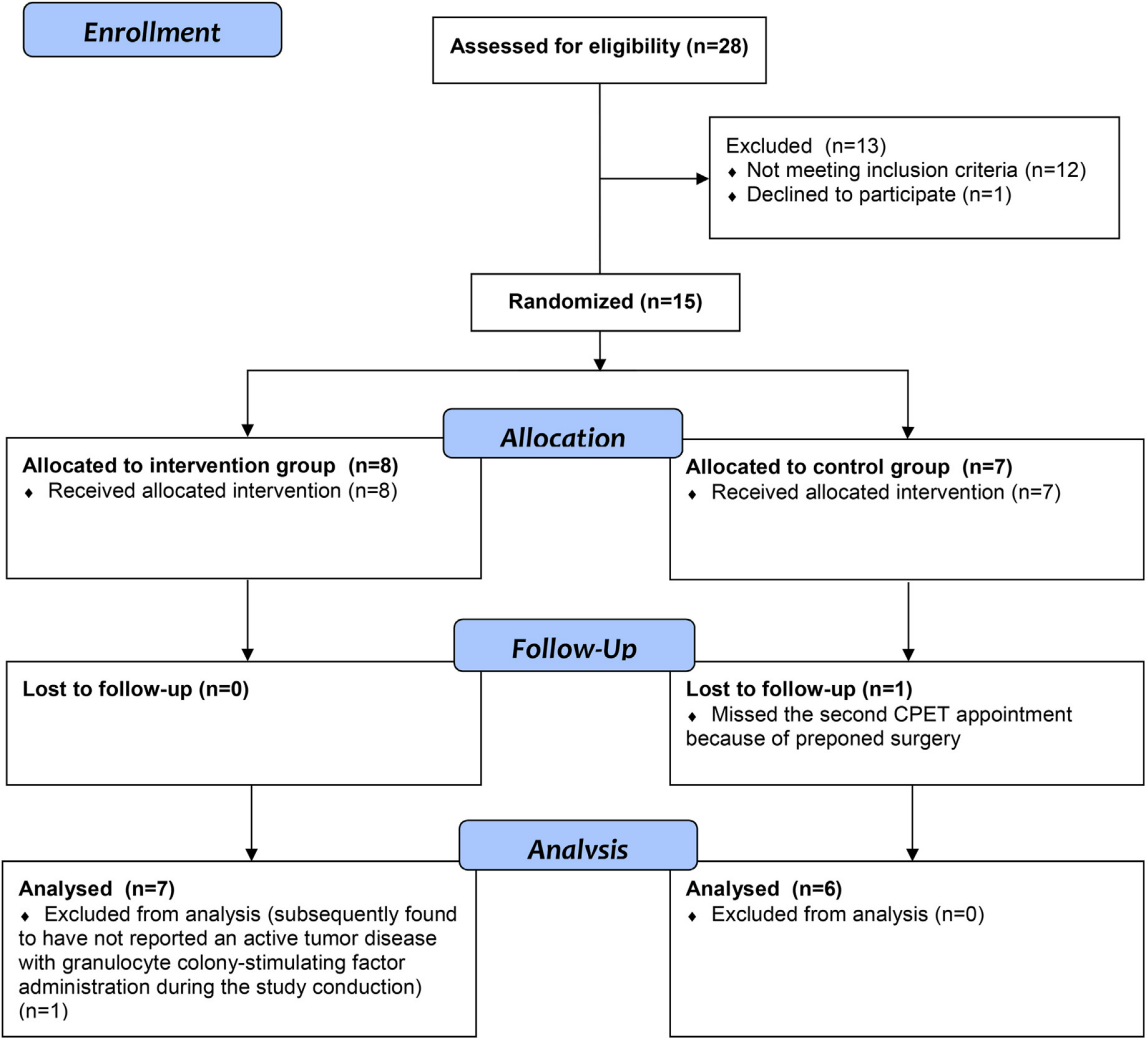
**CONSORT diagramme.** Study flow chart visualizing trial feasibility with a low drop-out rate between patients' trial participation consent and final analysis (total drop-out rate = 13.33%; n_drop out_ = 2/15).

The trial design envisaged a trial participant allocation into two parallel groups with equal randomisation (1:1). The random allocation sequence was determined via block randomisation with block sizes of four by drawing lots from a container, performed by a member of our study group not otherwise involved into the operational conduct of this trial (random allocation sequence generated by V.S., participants enrolled by S.L. and R.S., and participants assigned to the intervention by CJ.B. in adherence to the random allocation sequence). Participants were randomly assigned to either prehabilitation (n = 8) with vigorous-intensity exercise for four weeks or to the control group (standard care, no exercise, n = 7). Two participants were excluded from the study (one in each group) due to active comorbid tumor disease with granulocyte colony-stimulating factor administration or failure to attend an assessment. Therefore, per protocol analysis was undertaken with 13 patients (see Figure 1), including seven participants without reported cardiovascular risk factors or disease and six patients with preoperative comorbid disease, respectively.

This trial was conducted unblinded.

The work has been reported in line with the STROCSS (“Strengthening the reporting of cohort, cross-sectional and case-control studies in surgery”) criteria [21].

### 2.2 Study intervention: four-week preoperative exercise programme

Exercise programme designs are recommended to be described or compared according to the FITT (frequency, intensity, time and type) principle [22]. The small number of available previous trials applying a prehabilitation programme in the area of major non-cardiac surgery was taken into account for the design of this trial's exercise programme. Analysed by means of the FITT principle, literature data for the applied training frequency ranged from daily [23, 24, 25] to once a week [26] (with the majority of trials applying two to three sessions a week); targeted training intensity ranged from mild (40% of heart rate reserve) [27] to high (>80% of heart rate reserve) [28, 29]; training time ranged between 20 min [30] and 1 h per session [23, 31, 32, 33, 34]; training type varied from endurance training only [25, 26, 28, 29, 31, 35] to combinations of endurance, resistance and dedicated inspiratory muscle training [33], and total training programme duration ranged from two [33, 36] to nine weeks [24] (but most trials scheduled four to six weeks [25, 26, 27, 29, 31, 32, 34, 37]).

In consensus with the first clinical guideline including recommendations for prehabilitation programmes [38] and clinical feasibility, this study implemented a four-week preoperative exercise programme (prehabilitation) in the intervention group, comprised of two to three appointments per week for vigorous-intensity [39] interval training on cross-walkers (Milon Industries GmbH, Emersacker, Germany).

In the clinical setting of this study, there was only limited time preoperatively to conduct a training prior to surgery. In order to prevent trial participants' drop-outs, we scheduled nine to ten appointments in advance to maintain flexibility in case patients would have to skip one or two training sessions on short notice. At the end of the individualised four-week training phase, every participant was required to confirm a total number of eight or more exercise visits, and in fact only one intervention group participant finished 10 training sessions, another one finished 9 training sessions, and all other participants finished 8 training sessions. The duration of each training session was 34 min. Every training visit started with a 5-min warm-up, followed by six intervals of 4-min exercise phases on cross-walkers, with 1-min recovery phases in between each exercise phase. The cross-walkers used in this study continuously auto-adjusted their resistance to achieve the study participant's sub-maximum heart rate (set at 70–80% of each participant's maximum heart rate obtained at their baseline CPET) throughout the exercise phases. During recovery phases no resistance was applied by the cross-walkers and heart rate declined naturally without specific numeric targets.

The exercise sessions were conducted by a single certified sports scientist providing it face-to-face at the German Sports University Cologne. Intentionally, we placed high emphasis on training supervision in contrast to most previous studies, of which many had featured home-based unsupervised training and as a potential consequence had shown a clear tendency towards lower training adherence and prehabilitation programme efficacy [24, 25]. As with the Cardiopulmonary Exercise Testings, the full supervision of the four-week vigorous-intensity interval training provided patients with the ability to constantly express any complaints or adverse events. Additionally, at the beginning of each appointment, patients were asked for any adverse events occurred since the last appointment requiring medical consultancy/intervention or not. All collected data was then stored in a main study data excel sheet and classified according to Common Terminology Criteria for Adverse Events (CTCAE) scale (version 4.03) [40].

### 2.3 Outcome measures

#### 2.3.1 Cardiopulmonary exercise testing (CPET)

Baseline fitness level was assessed using standardised cardiopulmonary exercise testing (CPET) with a cycle ergometer (Custo med GmbH, Ottobrunn, Germany), spirometry analysis (Cortex Biophysik GmbH, Leipzig, Germany), continuous gas exchange analysis (oxygen consumption [$\dot{\text{V}}$O_2_, mL/kg/min] and carbon dioxide production [$\dot{\text{V}}$CO_2_, mL/kg/min]) and electrocardiogram monitoring for heart rate analysis (Custo med GmbH, Ottobrunn, Germany). Cardiopulmonary exercise testing was supervised by a medical doctor with expertise in cardiopulmonary resuscitation. Supervision also enabled patients constantly to express any complaints or adverse events.

CPET followed a ramp protocol with a multistage incremental step test protocol. After a 1-min rest measurement and a 3-min warm-up at 50 W power output at 45–55 revolutions per minute, the workload was increased by 25 W every 2 min until self-perceived exhaustion with unability to maintain a pedal cadence of 45–55 revolutions per minute on the cycle ergometer, cardiovascular or pulmonary distress, or fatigue occurred. Objective exhaustion was verified by a respiratory exchange rate >1.1. After peak exercise, all participants underwent a 3-min recovery phase. Oxygen consumption at anaerobic threshold (AT, mL/kg/min) was calculated utilizing the modified V-slope method of plotting the exhaled carbon dioxide production ($\dot{\text{V}}$CO_2_) against oxygen uptake ($\dot{\text{V}}$O_2_) with increasing workload, as described by Wasserman et al [41]. Peak $\dot{\text{V}}$O_2_ was defined as the maximum oxygen consumption achieved during the exercise test (provided by MetaSoft ® Studio version 4.8.2 and representing the peak value, not peak of an average of multiple breaths—even though, admittedly, we would recommend the latter, nowadays more commonly used method for future projects considering its potential variability reduction [42]).

#### 2.3.2 Endothelial progenitor cell analysis by flow cytometry

**Figure 2:**
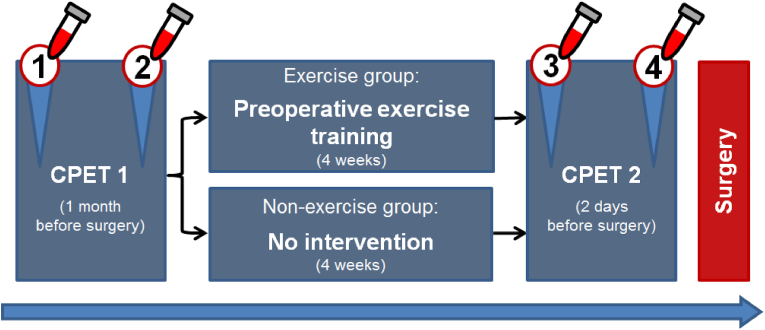
**Study design.** This trial applied a vigorous intensity interval training programme for four weeks preoperatively in patients randomly assigned to the exercise group. Cardiopulmonary Exercise Testing (CPET) was performed at baseline and repeated four weeks later to assess physical capacity. Blood samples for endothelial progenitor cell (EPC) measurement were collected after each CPET to investigate the acute cellular response to exhaustive exercise, and before the second CPET in order to measure the sustained cellular response to the four-week non-exhaustive exercise programme. Both exercise group and control group had equal representation of patients without reported cardiovascular disease and patients with comorbid disease.

For the evaluation of the effects of a four-week preoperative training on a) mature endothelial cells and b) EPC, we collected blood samples before and after both CPETs (first CPET prior to prehabilitation; second CPET after completion of prehabilitation) (see Figure 2).

In cell processing tubes, a diluted cell suspension was created from blood and phosphate-buffered saline (PBS), and layered above 15 mL Ficoll™-Paque (PAN-Biotech GmbH, Aidenbach, Germany). Centrifugation was performed at room temperature for 20 min at 2,400 revolutions per minute. After isolation, the mononuclear cells underwent a cleaning step and were then transferred into cryotubes containing 1.8 mL of freezing medium (fetal bovine serum with 10% DMSO). Microscopic cell counting of a representative 10 μl sample, stained with Trypan Blue, inside a “Neubauer improved” haemocytometer (Laboroptik GmbH, Friedrichsdorf, Germany) ensured optimal peripheral blood mononuclear cell (PBMC) concentrations, with between 10–20 million cells per cryotube. These PBMC samples then underwent gentle freezing inside isopropanol containers and a −80 degrees Celsius freezer before being stored in liquid nitrogen tanks until batch analysis.

In the absence of consensus on the precise surface marker definition of EPCs in response to exercise and training, we used an a priori approach to develop a working surface marker profile for putative EPCs in our patients. A panel of surface markers used widely in the literature for human EPC enrichment [43] and in previous studies by our group was selected. These markers included prominin-1 (PROM-1/CD 133), kinase insert domain receptor (KDR/Flk1/VEGF receptor 2/CD309), platelet endothelial cell adhaesion molecule (PECAM-1/CD31), and CD14 along with CD34 as a surrogate marker for stemness among these populations. This combination permits the isolation of EPCs while minimizing contaminant cells that share some of the characteristics of EPCs, such as blood monocytes (CD45 + CD14+/++). The CD45dim/- population was used to exclude the vast majority of hematopoietic cells. We sought subpopulations of cells within this parent population whose abundance changed following CPET or four weeks of training. Internal compensation was used in all analyses.

The batch analysis started with rapid thawing in a 37 degrees Celsius water bath, followed by three washing steps in FACS (“fluorescence activated cell sorting”) buffer with centrifugation in between (300g and 4 degrees Celsius) for 10 min. Before the last centrifugation, filtration through a 100-micron mesh was performed. After cell counting, 100 μL of labelling mixture were used per 10 million cells. The labelling included 3, 4, and 5 label-containing solutions for at least two replicates and all six labels (anti CD14, CD31, CD34, CD45, CD133, and CD309 monoclonal antibodies) for all samples. The incubation process was followed by a 1000-fold dilution, washing with centrifugation, and resuspension in further FACS buffer at 4 degrees Celsius.

Compensation controls were used for the analysis setup, with negative and single-label positive controls for compensation using beads and cells as required. Analysis and sorting were undertaken on the BD FACS Aria II (Stanford Shared FACS Facility, CA, USA) using the BD FACS Diva software. Analysis of the.fcs files was undertaken using Cytobank with Flow Self Organised Maps (FlowSOM), Cluster Identification, Characterisation and Regression (CITRUS), Barnes-Hut t-SNE algorithm cluster analysis (ViSNE), and Spanning-tree Progression Analysis of Density-normalised Events (SPADE) analysis [44]. All subpopulations were then interrogated and quantified using region of interest tools within Cytobank as part of standard flow analysis.

#### 2.3.3 Cluster-based analysis

Pre-CPET and post-CPET blood samples were used to perform “CITRUS” analysis (Cluster Identification, characterisation, and regression) with the goal to identify cell subpopulations that demonstrate recruitment patterns following CPET, given that single-episode exhaustive exercise (such as cardiopulmonary exercise testing) has been shown to increase levels in circulating EPCs in previous literature [11, 45].

The samples were divided into two batches for analysis. The Barnes-Hut implementation of the t-SNE algorithm (viSNE), paired with Cluster Identification, characterisation, and regression (CITRUS) were performed on the first batch to provide an a priori approach to the identification of novel, potentially clinically relevant subpopulations of cells within the peripheral circulation that changed in abundance in peripheral blood samples of some individuals following CPET and/or exercise training. The surface marker profiles of these subpopulations were then compared with existing EPC marker profiles within the literature to identify putative EPC populations. The relatedness and hierarchical structures of these clusters were further interrogated by FlowSOM (Self-Organising Map) to ensure the subpopulations had robust features and that clusters with low abundance were not excluded from the analysis. The surface marker profile of the most predictive cluster was then confirmed during the analysis of the second batch, and this definition was used as the basis for standardised FACS analysis using a combination of single and bi-dimensional gating. Quantitative analysis of EPC changes following exercise and training by conventional flow analysis ensured maximum ability to compare the findings with those within the literature. All flow and cluster-based analyses were performed using Cytobank.

#### 2.3.4 Mature endothelial cell investigation

While the main focus of this study was on EPC subpopulations, back-gating allowed for the identification of subpopulations and regions that enriched for putative endothelial cells within CD14-CD45-CD133-gated events by examining their CD31 expression. Circulating mature endothelial cells were defined as being within this group but strongly CD31 positive.

### 2.4 Statistical analysis

The primary endpoint was defined to be the effect of a four-week preoperative vigorous intensity interval training programme on physical capacity (as measured by oxygen consumption at anaerobic threshold and peak exercise). Secondary endpoints included the prehabilitation programme's effect on peripheral blood EPC recruitment and the incidence of postoperative complications (in particular cardiac events, pulmonary, wound healing and surgical complications requiring surgical revision, that were classified according to Clavien-Dindo score [46]).

Group homogeneity in patient characteristics and demographics was analysed via Shapiro-Wilk test of normality, Levene's test for equality of variances, and in accordance with their results, independent samples t-test, Wilcoxon or Mann-Whitney U test. The same methods were applied to investigate changes from pre-exercise levels in physical capacity parameters and cell subpopulations and the quantity of EPCs in response to the four-week preoperative exercise training programme. Correlation analysis was performed via Pearson correlation after ensuring metric scaling and normal distribution of the analysed data.

The level of significance was set at p ≤ 0.05 in all tests. All statistical analyses were performed in SPSS ® Statistics (Version 23.0.0.0; IBM Corp., Armonk, USA).

### 2.5 Patient and public involvement

Patients or the public were not involved in the design, conduct, reporting, or dissemination plans of our research.

## 3 Results

Fifteen patients scheduled for abdominal laparotomy for incontinence surgery that met the eligibility criteria were enrolled in this study. Patient recruitment lasted from 01st December 2014 until 30th November 2016.

With the intention to ensure patient population homogeneity, only a single, specific surgical technique was applied to all study participants. Therefore, patient enrollment was ended after the planned two-year recruitment period and due to the change in gynaecological staff introducing new surgical techniques that led to incomparability of the study subpopulations.

One patient (non-exercise group) was excluded from data analysis due to missing the second CPET appointment because of preponed surgery. A second patient (exercise group) initially enrolled was subsequently found out to have not reported her active tumor disease with granulocyte colony-stimulating factor administration during the study conduction and was therefore excluded from the analysis. Patient baseline characteristics are summarised in Table 1. There was no significant difference in both groups at baseline.

**Table 1:** Baseline characteristics indicate sufficiently matched groups.

	Total sample n = 13	Intervention group n = 7	Control group n = 6	p-value
**Female**	13/13	7/7	6/6	>0.999
**Age [Years]**	59.62 ± 10.42	57.29 ± 8.67	62.33 ± 12.40	0.445
**Weight [kg]**	67.00 ± 8.90	66.43 ± 6.66	67.67 ± 11.66	0.815
**Height [cm]**	167.54 ± 5.27	167.57 ± 4.65	167.50 ± 6.38	0.982
**BMI [kg/m**^**2**^**]**	23.95 ± 3.62	23.81 ± 3.78	24.12 ± 3.77	0.888
**Waist circumference [cm]**	89.15 ± 11.60	88.71 ± 12.18	89.67 ± 12.03	0.890
**Arterial Hypertonia**	3/13	2/7	1/6	0.731
**Hyperlipidemia**	5/13	3/7	2/6	0.836
**Statin treatment**	4/13	3/7	1/6	0.445
**Abdominal Obesity**	6/13	3/7	3/6	0.836
**Smoker**	1/13	0/7	1/6	0.628
**Diabetes Mellitus (IDDM)**	1/13	1/7	0/6	0.731
**Diabetes Mellitus (NIDDM)**	0/13	0/7	0/6	>0.999
**Coronary Heart Disease**	0/13	0/7	0/6	>0.999
**Chronic Heart Failure**	0/13	0/7	0/6	>0.999
**Chronic Renal Failure**	0/13	0/7	0/6	>0.999
**ASA ≥2**	6/13	3/7	3/6	0.836
**rCRI ≥2**	0/13	0/7	0/6	>0.999
$\dot{\text{V}}$**O**_**2**_**peak [mL/min/kg]**	29.62 ± 9.81	31.00 ± 9.80	28.00 ± 10.49	0.605
**Anaerobic threshold**	17.08 ± 5.42	17.14 ± 5.52	17.00 ± 5.83	0.965
**[mL/min/kg]**				

Numeric values are given as mean ± standard deviation.

BMI = Body mass index.

IDDM = Insulin-dependent diabetes mellitus.

NIDDM = Non-insulin-dependent diabetes mellitus.

rCRI = Revised Cardiac Risk Index.

ASA = American Society of Anesthesiologists Physical Status Classification.

$\dot{\text{V}}$O_2_ peak = Maximum oxygen consumption achieved during the exercise test.

With regards to the intervention, namely the four-week vigorous-intensity interval training, as well as both Cardiopulmonary Exercise Testings, no harms (with respect to the CONSORT definition [47]) were reported by the patients of this trial. Furthermore, no falls or injuries were observed during exercise.

Regarding events within the inpatient period perioperatively, the complete patient file of study patients was daily investigated for adverse events requiring medical intervention or surgical revision until postsurgical hospital discharge, but none were observed.

In particular, no postoperative complications of relevance to this study (such as cardiac events, pulmonary, wound healing or surgical complications) were reported. No patient required intensive care unit administration and the average duration of the hospital stay was 5.92 days (intervention group (mean [range]) 5.86 [5 to 7] days vs. control group (mean [range]) 6.00 [5 to 8] days).

There was no follow-up of patients after postsurgical hospital discharge. The last enrolled study participant completed our study on 25th May 2016.

### 3.1 Prehabilitation increases physical capacity but has no effect on circulating mature endothelial cell levels

Trial participants from both groups started with similar physical capacity at baseline according to their CPET results quantified by peak $\dot{\text{V}}$O_2_ (intervention group (mean [range]) 31.00 [20–42] mL/min/kg vs. control group (mean [range]) 28.00 [17–40] mL/min/kg; p = 0.605, two-tailed independent samples t-test) and anaerobic threshold (intervention group (mean [range]) 17.14 [9–25] mL/min/kg vs. control group (mean [range]) 17.00 [11–25] mL/min/kg; p = 0.965, two-tailed independent samples t-test).

For the verification of objective exhaustion of each trial participant during each CPET a respiratory exchange rate >1.1 was requested. This threshold was surpassed by all participants in both CPETs. (CPET1 intervention group (mean [range]) 1.18 [1.11–1.25] vs. CPET1 control group (mean [range]) 1.19 [1.14–1.28]; CPET2 intervention group (mean [range]) 1.17 [1.12–1.26] vs. CPET2 control group (mean [range]) 1.18 [1.11–1.25]).

In response to the four-week preoperative exercise programme, the comparison between intervention group and control group showed a statistically significant increase in both anaerobic threshold (Δ CPET2-CPET1 intervention group (mean [range]) 1.71 [−2 to 8] mL/min/kg vs. Δ CPET2-CPET1 control group (mean [range]) −1.83 [−4 to 1] mL/min/kg; p = 0.042, two-tailed independent samples t-test) and maximum oxygen uptake ($\dot{\text{V}}$O_2_peak; Δ CPET2-CPET1 intervention group (mean [range]) 1.71 [0 to 4] mL/min/kg vs. Δ CPET2-CPET1 control group (mean [range]) −1.67 [−4 to 0] mL/min/kg; p = 0.002, two-tailed independent samples t-test).

Comparing blood samples from before first (timepoint one = T1) and second (timepoint three = T3) CPET, no statistically significant changes in circulating mature endothelial cells (ECs: CD14–45–133-31+) were identified (Δ T1–T3 intervention group (mean [range]) -284 [−1131 to +85] events/μL vs. Δ T1–T3 control group (mean [range]) +1313 [−136 to +7014] events/μl; p = 0.138, two-tailed Mann-Whitney U test).

### 3.2 A priori identified EPC subpopulation shows significant recruitment after prehabilitation

**Figure 3:**
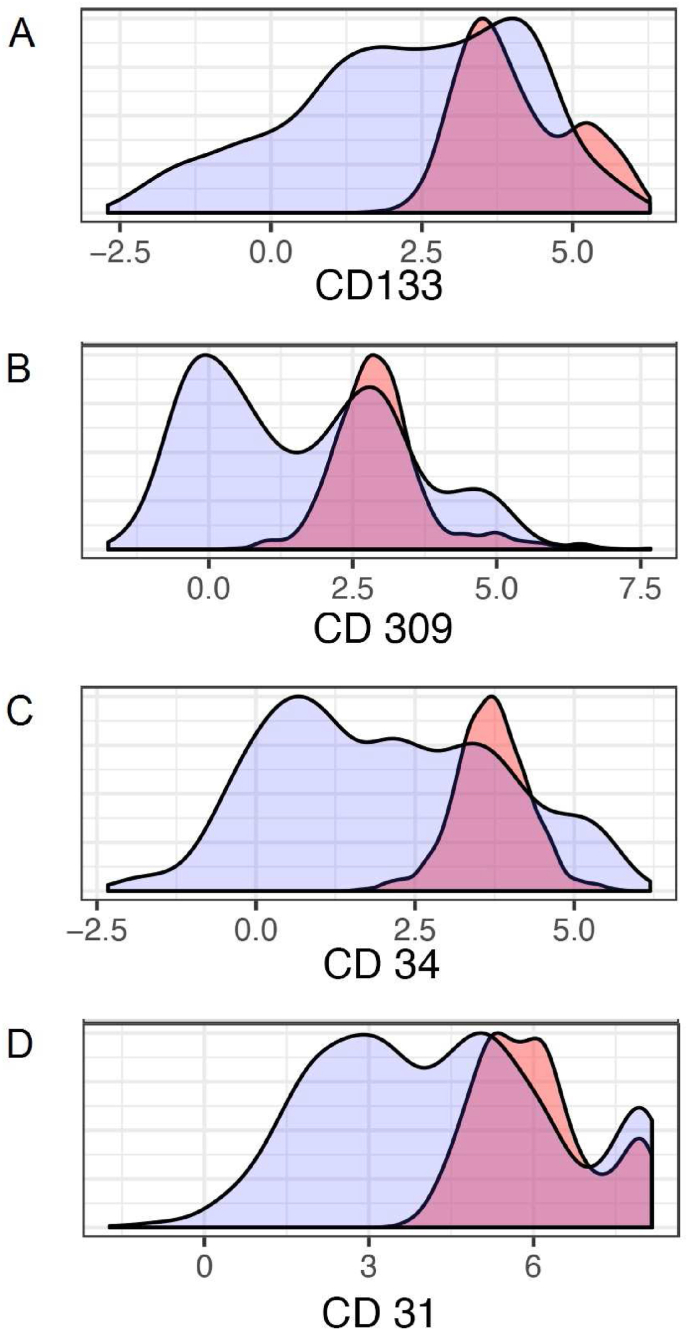
**Cell surface marker expression in the identified, novel training-responsive subpopulation.** Cell surface marker labelling profile for our putative endothelial progenitor cell subpopulation (pink) vs. the general pool of cells within the CD45dim parent population (blue). Each plot represents the expression of a specific cell surface marker as follows: **A.** Staining for CD133 expression. **B.** Staining for CD309 expression. **C.** Staining for CD34 expression. **D.** Staining for CD31 expression.

**Figure 4:**
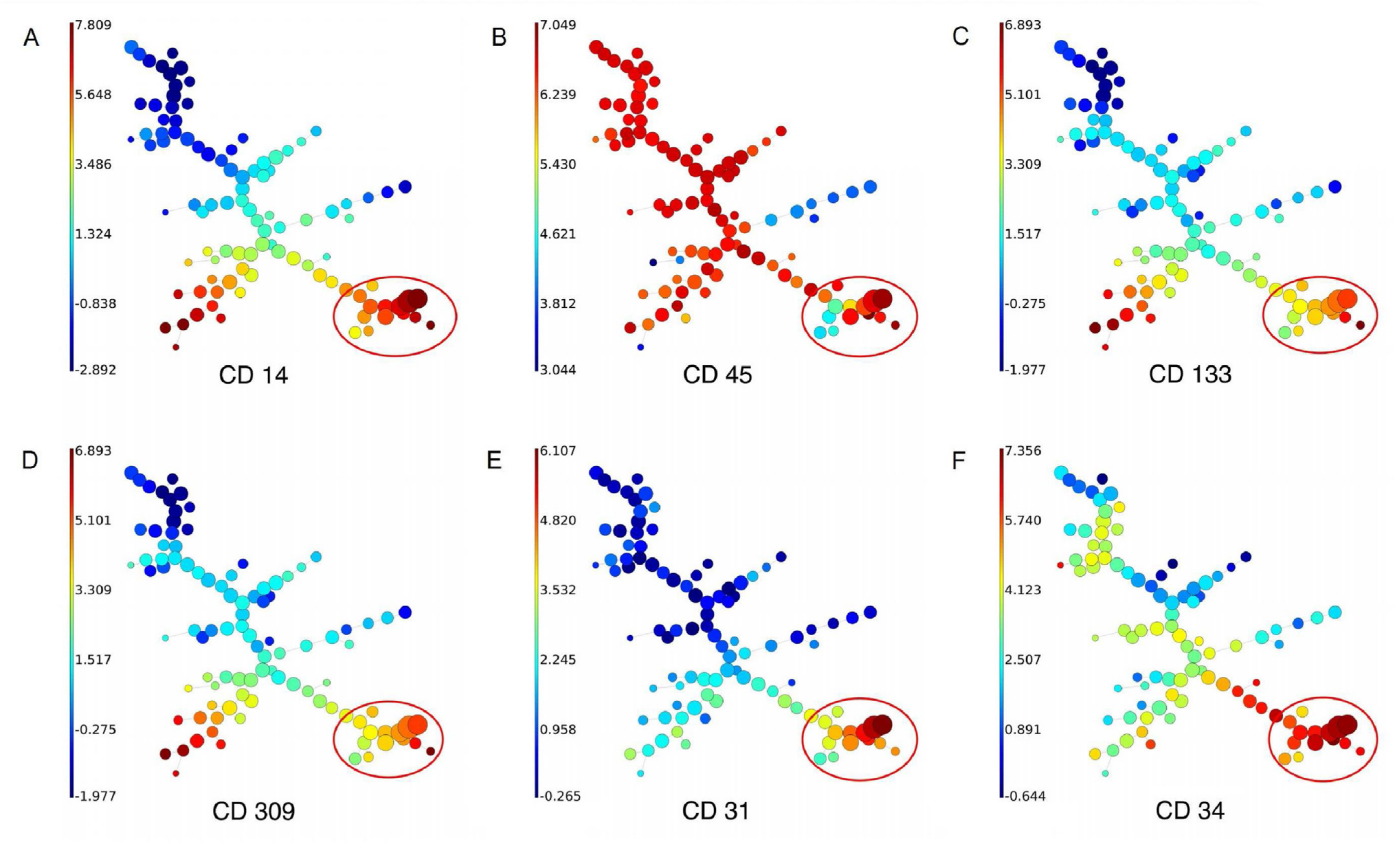
**FlowSOM analysis**. Each plot represents the expression of a specific cell surface marker (the expression extent is color-coded, as reflected by the relative scale color intensity bar on the left side of each plot) as follows: **A.** CD14 expression. **B.** CD45 expression. **C.** CD133 expression. **D.** CD309 expression. **E.** CD31 expression. **F.** CD34 expression. Specific clusters demonstrate similar features (marker profiles) and relatedness to other clusters like branches of a tree. The bottom right in each expression plot (circled red) shows the subpopulations that display an endothelial progenitor cell (EPC) phenotype. According to this analysis, the subpopulation of interest of this publication is closely associated with subpopulations that become more endothelial cell-like in their phenotype (increased expression of CD31 and reduced expression of putative EPC/progenitor markers including CD133 and CD309) suggesting that the subpopulation of interest is closely related to endothelial cells as potential progenitors.

Citrus (cluster identification, characterization, and regression) was used to test for subpopulations of cells/clusters that changed in response to CPET or training. The minimum cluster size was set to 5; equal sampling was applied to each file, resulting in 153 events being used per file. The cross-validation folds were set to 5; the false discovery rate was set to 1, and abundances were used for clustering characterization. When applied to the flow results from the first of two batch analyses, this produced 29 distinct subpopulations, whose marker profiles were compared against known profiles for endothelial progenitor cells. Of these 29 subpopulations, many were positive for EPC markers, but the subpopulation demonstrating the most significant changes in abundance was chosen as our working definition for EPCs in our study (CD45dim/CD14dim/CD133+/CD309+/CD34+/CD31+), and this result was validated in the second batch analysis (see Figure 3). Both Citrus and FlowSOM demonstrated distinct clusters and progeny that became progressively more endothelial in their phenotype, in keeping with EPC populations (see Figure 4).

**Figure 5:**
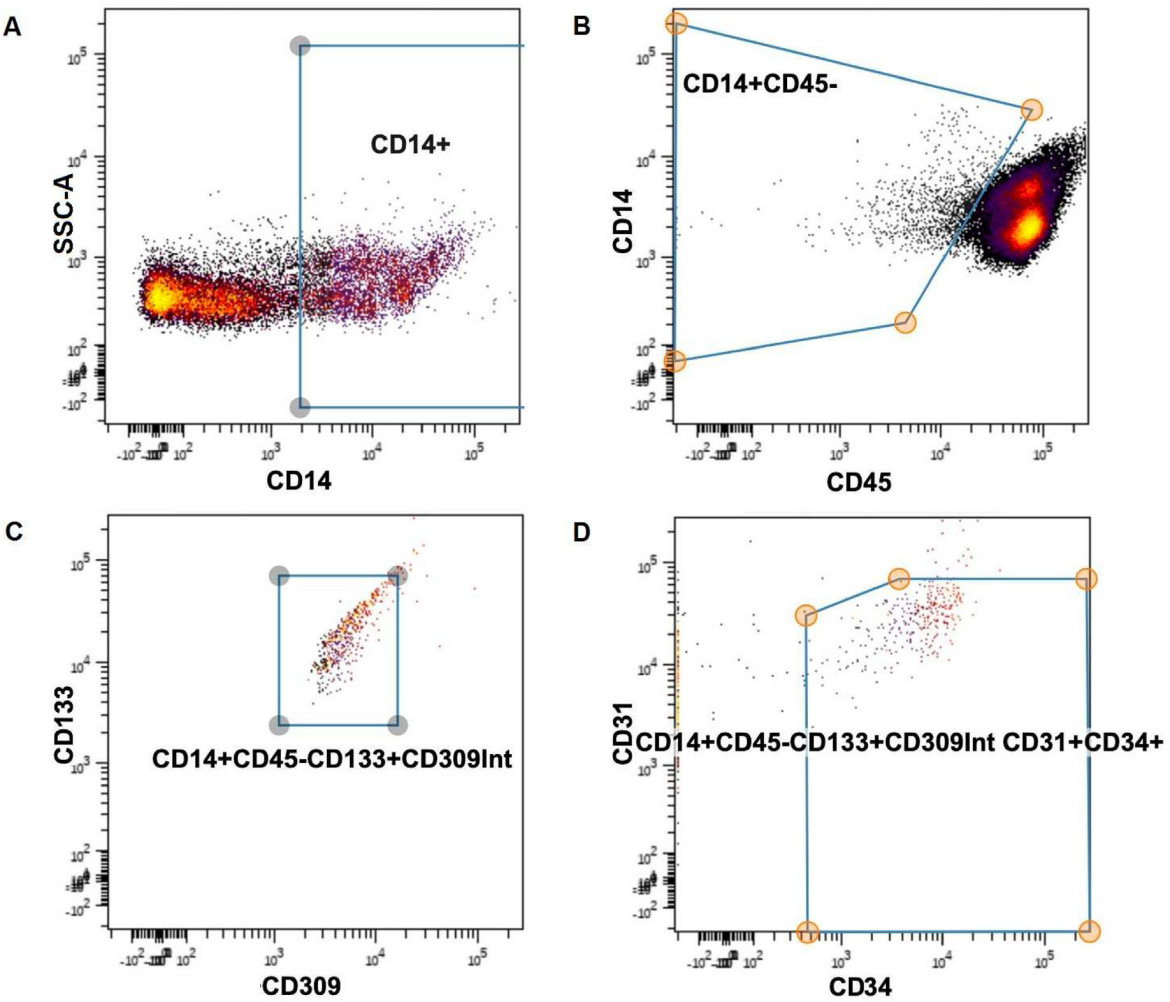
**Flow cytometry gating strategy.** The consecutive order of Plots A, B, C and D reflects the gating strategy used to isolate the endothelial progenitor cell (EPC) population based on the marker profile established by *a priori* analysis. This enriches for a novel EPC subpopulation. Parameters were plotted on the X and Y axis of each plot, as follows: **A.** CD14 expression (X axis) and Side Scatter Area “SSC-A” (Y axis). **B.** CD45 expression (X axis) and CD14 expression (Y axis). **C.** CD309 expression (X axis) and CD133 expression (Y axis). **D.** CD34 expression (X axis) and CD31 expression (Y axis).

**Figure 6:**
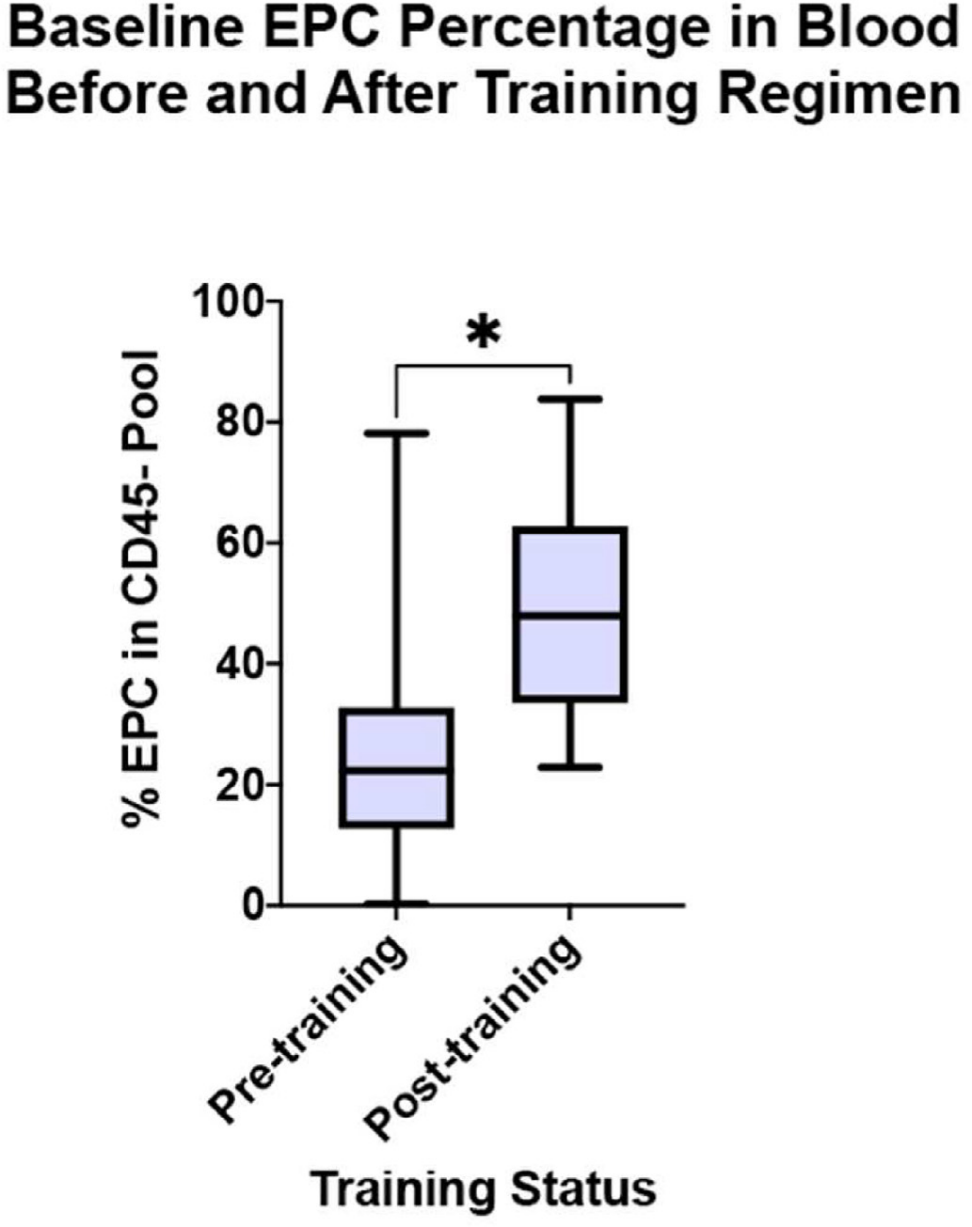
**EPC proportion changes in response to training.** A statistically significant increase in the baseline percentage of EPCs in the CD45 negative parent population within the circulating peripheral blood is demonstrated following four weeks of exercise interval training, suggesting a cellular basis for the effect of training as part of prehabilitation (p = 0.016, Wilcoxon matched signed-rank test). No such effect was seen in the non-trained group (p = 0.22).

The multidimensional gating strategy derived from this analysis is shown in Figure 5. Using this gating strategy, our putative EPC subpopulation demonstrated a statistically significant increase as a proportion of CD45-cells within peripheral blood at baseline following exercise interval training (51.37% of CD45-pool, 95% CI 22.88%–83.79% vs. 27.07%, 95% CI 0.3%–78.11%; p = 0.016, Wilcoxon matched signed-rank test; Figure 6). The non-training group did not demonstrate any significant change in their EPC percentage within the CD45-pool (p = 0.22).

Containing to improve the signal-to-noise-ratio to detect changes within small subpopulations of cells, the investigation of the putative EPC subpopulation happened as a proportion of the more refined pool of circulating CD45-cells rather than total mononuclear cells. It was validated that the CD45-pool did not make up more/less of the total PBMC pool over the training period (no significant changes were detectable, especially not within the intervention group [intervention group: p = 0.612 vs. control group: p = 0.116], comparing CD45-event counts from time point 1 and 3 via Wilcoxon matched signed-rank test).

Further analysis was performed regarding the association of functional capacity improvement as a result of the (non-)training phase (measured by $\dot{\text{V}}$O_2_ peak or anaerobic threshold) and the increase in the CD45dim/CD14dim/CD133+/CD309+/CD34+/CD31 + subpopulation as a proportion of circulating CD45-cells (via Pearson correlation). The results showed a positive correlation, but did not reach statistical significance (see Supplementary Table 1).

### 3.3 A four-week prehabilitation programme does not lead to significantly improved mobilisation of the a priori identified EPC subpopulation in response to a subsequent acute stress event

Changes in the CD45dim/CD14dim/CD133+/CD309+/CD34+/CD31 + subpopulation as a proportion of circulating CD45-cells were observable in the course of each CPET, as shown in Supplementary Table 2. Nevertheless, neither the comparison between ΔT1-T2 and ΔT3-T4 within each group (particularly the intervention group), nor the “inter-group” comparison of ΔT1-T2 or ΔT3-T4 resulted in differences of statistical significance (see Supplementary Table 2).

## 4 Discussion

Our proof-of-concept clinical trial found that prehabilitation with a four-week exercise interval training programme is feasible, improves physical capacity, and leads to an increased circulatory number of endothelial progenitor cells in patients scheduled for laparotomy.

For the longest time, there seems to have been a silent paradigm that people who have a surgically reversible disorder should undergo surgery as quickly as possible and afterward regain physical strength through rehabilitation. This notion of not preparing for surgery with exercise might have been derived from the intuitive approach of organisms in general to rest when affected by a disorder, most likely to avoid a worsening state. This hypothesis is consistent with a decline in oxygen consumption at the anaerobic threshold and peak exercise observed in this trials’ control group, suggesting that these patients became increasingly less fit over time, probably due to their underlying disease and deconditioning from a lack of exercise.

During the last decade, scientists and doctors have begun to put more focus on prehabilitation. While there has been a small number of clinical trials investigating the efficacy of prehabilitation in the area of major non-cardiac surgery [23, 24, 25, 26, 27, 28, 29, 30, 32, 33, 34, 35, 36, 37, 48, 49], cellular mechanisms affected by preoperative exercise have not yet been revealed.

In an attempt to widen the horizon of exercise-based prehabilitation to cellular effects, we sought to stimulate peripheral blood recruitment of endothelial progenitor cells, a key cell line in endothelial regeneration in adults, through prehabilitation. After having shown acute effects earlier [11], we now found an increased circulatory number of EPCs after four weeks of exercise. We suggest that exercise may improve the regenerative response that follows the traumatic insult of surgery, and endothelial regeneration may contribute to the reduction of complications observed in recent prehabilitation trials [26, 50].

The correct surface marker definition and the very existence of circulating endothelial progenitor cells as a discrete progenitor population has been debated for some time since the identification of EPCs as a bone marrow-derived progenitor cell population by Asahara over two decades ago [51]. Multiple surface marker definitions of EPCs have been published within the literature [43]. The marker profile identified seems to change depending on the animal studied, the in-vitro or in-vivo nature of the study, and the causative stimulus for the EPC recruitment, among many variables. Rather than test multiple potential definitions in the hope of finding one that will match our data, we used an a priori approach. Given that cardiopulmonary exercise testing (CPET) is associated with recruitment of EPCs into the peripheral circulation, we examined for subpopulations of cells that demonstrated recruitment patterns following CPET through the use of pre-CPET and post-CPET blood samples. CITRUS (cluster identification, characterisation, and regression) was developed to permit the automated identification and stratification of subpopulations in multidimensional cytometry where known or expected changes in subpopulations are anticipated [52]. A novel subpopulation of progenitor cells with a cell surface marker profile consistent with EPC's was therefore derived, and this was then tested for changes in recruitment in response to the training stimulus, thereby expanding on our previous studies that examined the impact of CPET alone.

While the underlying mechanisms leading to the observable increase in the number of circulating EPCs in response to a four-week vigorous-intensity interval training may go beyond simple cell mobilisation from e.g. bone marrow (which has been the suggested mechanism for short-term circulating EPC level increases seen after single episodes of exhaustive exercise [11, 45]), and also include less prompt effects, such as improved circulatory conditions with reduced apoptotic stimuli and therefore improved EPC survival, our clinical trial strengthens the proposition that circulating EPC levels are modifiable in response to specific stimuli, also including non-pharmacologic interventions such as exercise and training, and provides mechanistic support at a cellular level for the potential benefits of prehabilitation, reduced surgical complications, and improved health with exercise in general.

## 5 Limitations

Being a proof-of-concept pilot study, this trial lacks statistical power to prove that the acquired EPC recruitment leads to desired clinical implications like a reduction in postoperative complications, length of hospital stay, or intensive care unit admissions. Most prehabilitation studies are underpowered with these endpoints and only a few trials were able to show an effect of exercise on postoperative complications [26]. It seems fair to assume that a selection of patients with more comorbidities within our cohort may have demonstrated higher efficacy of prehabilitation on outcome. In order to successfully address this issue, patients with higher ASA (“10.13039/100013331American Society of Anesthesiologists Physical Status Classification”) scores will be enrolled in future studies, as supported by the findings of a recent systemic review [53].

Even though CITRUS analysis detected a novel subpopulation of progenitor cells with a cell surface marker profile consistent with EPCs that shows responsiveness to the training stimulus applied in this study, and FlowSOM analysis indicated close relatedness of this subpopulation to mature endothelial cells suggesting putative progeny, this study does not provide evidence if the mentioned cell subpopulation does indeed exhibit functionality (in vitro or in vivo) as expected from endothelial progenitor cells (e.g. paracrine proangiogenic functionality to promote vascular regeneration, and differentiation into mature endothelial cells [54]).

Therefore, functional characterisation of our novel subpopulation of putative EPCs (including cell culture analysis and other methodology) warrants further ongoing research to validate these positive findings at a cellular level.

## 6 Conclusion

This proof-of-concept pilot study provides a feasible and effective prehabilitation concept within a framework of vigorous-intensity interval training and a potential mechanism for improved cellular regenerative response to the trauma of surgery, which may help reduce the incidence of postoperative complications.

## Declarations

### Author contribution statement

Claus Juergen Bauer, Robert Schier, Michael Findlay: Conceived and designed the experiments; Performed the experiments; Analyzed and interpreted the data; Contributed reagents, materials, analysis tools or data; Wrote the paper.

Philipp Zimmer, Sebastian Ludwig, Christina Koliamitra: Conceived and designed the experiments; Performed the experiments; Contributed reagents, materials, analysis tools or data.

Volker Schick: Conceived and designed the experiments; Performed the experiments; Wrote the paper.

Geoffrey C. Gurtner: Conceived and designed the experiments; Analyzed and interpreted the data; Wrote the paper.

Bernhard Riedel: Conceived and designed the experiments; Analyzed and interpreted the data; Contributed reagents, materials, analysis tools or data; Wrote the paper.

### Funding statement

This work was supported by Deutsche Forschungsgemeinschaft [491454339].

### Data availability statement

The data that has been used is confidential.

### Declaration of interest’s statement

The authors declare no conflict of interest.

### Additional information

The clinical trial described in this paper was registered at “German Clinical Trials Register (DRKS)” under the registration number “Registration ID: DRKS00000527”.

## Appendix A Supplementary data

04_Suppl._Suppl.Tables [UNCHANGED]_V3 04_Suppl._Suppl.Tables [UNCHANGED]_V3

Revised_CONSORT_Checklist_V02 Revised_CONSORT_Checklist_V02
