# Supplementary material for: Preoperative exercise induces endothelial progenitor cell mobilisation in patients undergoing major surgery – A prospective randomised controlled clinical proof-of-concept trial
Source: Heliyon. 2022 Sep 23;8(9):e10705. doi: 10.1016/j.heliyon.2022.e10705 (PMC9529507; doi:10.1016/j.heliyon.2022.e10705)
Supplement: 04_Suppl._Suppl.Tables [UNCHANGED]_V3 [file mmc1.doc]

|  | **V̇O_2_peak**  ∆ T1-T3 | | |  | | **Anaerobic threshold**  ∆ T1-T3 | | |  | |
| --- | --- | --- | --- | --- | --- | --- | --- | --- | --- | --- |
|  | Pearson Correlation | p- value^§^ |  | | Pearson Correlation | | p- value^§^ |  | |  |
|  |  |  |  | |  | |  |  | |  |
| **CD45dim/CD14dim/CD133+/CD309+/CD34+/CD31+ subpopulation as a proportion of CD45- cells**  ∆ T1-T3 |  |  |  | |  | |  |  | |  |
| Total sample (n=13) | 0.412 | 0.162 |  | | 0.326 | | 0.278 |  | |  |
| Intervention group( n=7) | 0.331 | 0.468 |  | | -0.045 | | 0.924 |  | |  |
| Control group (n=6) | 0.053 | 0.921 |  | | 0.551 | | 0.257 |  | |  |
|  |  |  |  | |  | |  |  | |  |

**Supplementary Table 1.** **Association of physical capacity improvement and the increase in the proportionate CD45dim/CD14dim/CD133+/CD309+/**

**CD34+/CD31+ subpopulation within the circulating CD45- pool.**

Pearson correlation revealed predominantly positive correlations between changes in physical capacity measures and changes of the proportionate CD45dim/CD14dim/CD133+/CD309+/CD34+/CD31+ subpopulation within the circulating CD45- pool that occurred in the course of the (non-)training phase, but none of these correlations reached statistical significance.

^§^ Significance, 2-tailed

|  | **Intervention group n=7** |  | **Control group**  **n=6** |  | **p-value**^§^ |
| --- | --- | --- | --- | --- | --- |
| **∆ T1-T2**  CD45dim/CD14dim/CD133+/CD309+/CD34+/CD31+ subpopulation as a percentage proportion of CD45- cells | 2.27±18.75 |  | 6.02±9.62 |  | 0.668 |
|  |  |  |  |  |  |
| **∆ T3-T4**  CD45dim/CD14dim/CD133+/CD309+/CD34+/CD31+ subpopulation as a percentage proportion of CD45- cells | -9.27±29.15 |  | 1.64±30.54 |  | 0.524 |
|  |  |  |  |  |  |
| **p-value**^§^ | 0.468 |  | 0.770 |  |  |

**Supplementary Table 2.** **Changes in the proportionate CD45dim/CD14dim/CD133+/CD309+/CD34+/CD31+ subpopulation within the circulating CD45- pool in the course of each Cardiopulmonary Exercise Testing.**

This tables illustrates all changes in the proportionate CD45dim/CD14dim/CD133+/CD309+/CD34+/CD31+ subpopulation within the circulating CD45- pool for both Cardiopulmonary Exercise Testings (∆ T1-T2 = change in the course of CPET 1; ∆ T3-T4 = change in the course of CPET 2) and both patient groups. Reading the lines of the table horizontally or the table columns vertically, different constellations can be compared (comparison between ∆ T1-T2 and ∆ T3-T4 within each group, or “inter-group” comparison of ∆ T1-T2 or ∆ T3-T4) and p-values are shown accordingly.

^§^ Significance, 2-tailed

Numeric values are given as mean ± standard deviation.
